# Both light and soil moisture affect the rhizosphere microecology in two oak species

**DOI:** 10.3389/fmicb.2025.1506558

**Published:** 2025-02-03

**Authors:** Jinshuo Lian, Keke Cai, Aijing Yin, Yuan Yuan, Xinna Zhang, Chengyang Xu

**Affiliations:** ^1^The Key Laboratory for Silviculture and Conservation of Ministry of Education, Key Laboratory for Silviculture and Forest Ecosystem of State Forestry and Grassland Administration, Research Center for Urban Forestry, Research Center of Deciduous Oaks, Beijing Forestry University, Beijing, China; ^2^State Key Laboratory of Efficient Production of Forest Resources, School of Ecology and Nature Conservation, Beijing Forestry University, Beijing, China

**Keywords:** light, soil moisture, *Quercus variabilis*, *Quercus dentata*, rhizosphere microecology

## Abstract

Understanding the mechanisms by which seedlings respond to light and water regulation, as well as studying the response of rhizosphere microecology to drought stress, are crucial for forest ecosystem management and ecological restoration. To elucidate the response of the rhizosphere microecology of *Quercus dentata* and *Quercus variabilis* seedlings to water and light conditions, and to clarify how plants modulate the structure and function of rhizosphere microbial communities under drought stress, we conducted 12 water-light gradient control experiments. These experiments aimed to offer scientific theoretical support for the dynamic changes in rhizosphere soil enzyme activities and microbial community compositions of these two oak species under varying light and moisture conditions, and subsequently assist in the future breeding and cultivation efforts. The results are summarized as follows: (1) The activities of cellulase, urease, and chitinase in the rhizosphere soil of *Q. dentata* and *Q. variabilis* were significantly influenced by water and light treatments (*p <* 0.05). Urease was particularly sensitive to light, while sucrase exhibited sensitivity to light in *Q. dentata and* no significant difference in *Q. variabilis*. (2) Compared to *Q. dentata*, the rhizosphere bacteria of *Q. variabilis* demonstrated greater adaptability to drought conditions. Significant differences were observed in the composition of microorganisms and types of fungi in the rhizosphere soil of the two *Quercus* seedlings. The fungal community is significantly influenced by light and moisture, and appropriate shading treatment can increase the species diversity of fungi; (3) Under different water and light treatments, the rhizosphere soil microbial composition and dominant species differed significantly between the two *Quercus* seedlings. For instance, *Streptomyces*, *Mesorhizobium*, and *Paecilomyces* exhibited significant variations under different treatment conditions. Specifically, under L3W0 (25% light, 75–85% moisture) conditions, *Hyphomonadaceae* and SWB02 dominated in the *Q. dentata* rhizosphere, whereas Burkholderiales and Nitrosomonadaceae were prevalent in the *Q. variabilis* rhizosphere. Overall, the rhizosphere microecology of *Q. dentata* and *Q. variabilis* exhibited markedly distinct responses to varying light and water regimen conditions. Under identical conditions, however, the enzyme activity and microbial community composition in the rhizosphere soil of these two oak seedlings were found to be similar.

## Introduction

Seedlings constitute the fundamental cornerstone of natural regeneration within forest ecosystems, actively participating in and propelling the intricate succession process from establishment to maturity (Zhao et al., 2025). They serve as vital replenishers, counterbalancing tree mortality stemming from both natural and anthropogenic causes, thereby preserving the continuity and resilience of forest ecosystems. In the context of forest restoration endeavors, the complementary dynamic and functional attributes of seedlings hold paramount importance, not only facilitating vegetation recovery but also safeguarding biodiversity (Chen et al., 2018). Furthermore, as a pivotal microbial habitat, the soil environment exerts profound influences on the structure and diversity of microbial communities (Rasche et al., 2011; Richter et al., 2018). This intricate ecosystem encompasses a symphony of nutrients, enzymes, and microorganisms, all intricately intertwined in their influence on plant growth and development (Jang et al., 2020; Xiao D. et al., 2015; Xiao Y. et al., 2015). Notably, soil properties such as pH, texture, moisture content, temperature, organic carbon levels, and nutrient availability play pivotal roles in shaping the composition and diversity of soil microbial communities (Brockett et al., 2012; Cookson et al., 2007; Rousk et al., 2010). These factors, in turn, contribute to the overall health and functioning of forest ecosystems, underscoring the intricate interplay between seedlings, soil, and microbial communities in the grand tapestry of forest restoration and conservation. The rhizosphere environment is an active region for information and material exchange between plants, microorganisms and soil (Lin et al., 2020). Rhizosphere microorganisms have a significant influence on the biochemical cycling process in soil, and they play a crucial role in the health and development of roots (Birt et al., 2022). In terms of rhizosphere microecology, previous studies focused on the effects of crop patterns such as crop rotation, continuous cropping and co-cropping on rhizosphere microecology (Liu et al., 2024; Huang and Li, 2024; Yin et al., 2024), effects of different diseases on plant rhizosphere microecology (Liu, 2023; Zhang et al., 2023), effects of elements required for growth and biological reagents (hormones) on rhizosphere microecology (Wang et al., 2024; Li et al., 2024; Han et al., 2024), lack of external light and moisture conditions on the influence of research.

Soil enzymes represent a crucial indicator of soil microecology, facilitating an array of biochemical reactions that underpin essential processes such as organic matter decomposition and the promotion of carbon, nitrogen, phosphorus, sulfur, and other nutrient cycles. By assessing soil enzyme activity, we can gain indirect insights into soil quality, thereby serving as a vital metric for evaluating soil health and fertility (Lu and Li, 2020). Soil microorganisms, another indispensable element of soil ecosystems, contribute significantly to element cycling, organic matter decomposition, rhizosphere immunity enhancement, soil fertility improvement, and environmental health maintenance (Zhu Y. G. et al., 2021; Huang et al., 2024). Their diversity is intricately influenced by factors such as vegetation composition, soil type, temperature, moisture content, and management practices (Zhou and Lei, 2007). Soil fungi, a vital component of the microbial system, occupy a pivotal role in material circulation and energy flow within soil ecosystems (Zhao L. N. et al., 2020; Tiwari et al., 2021). Similarly, bacterial communities drive soil ecosystem restoration, significantly contributing to ecological restoration, soil fertility enhancement, and water conservation efforts (Yuan and Zhang, 2024; Shi et al., 2024).

Extensive research underscores the profound impact of rhizosphere microbial quantity, diversity, metabolic activity, and interactions on plant health. These microorganisms can either bolster plant resistance and growth or, conversely, act as pathogens, thereby modulating plant health outcomes (Berendsen et al., 2012; Sun et al., 2015; Terhonen et al., 2019). Investigations into rhizosphere microecology have delved into various influencing factors, with Chen Lanlan et al. comprehensively reviewing plant responses to drought stress, encompassing plant morphology, physiological molecular mechanisms, and rhizosphere microbial community dynamics (Chen et al., 2024). This work provides a solid theoretical foundation for future rhizosphere microecology research.

Furthermore, Shen Xintao has illuminated the mechanisms underlying rhizosphere microbial regulation of plant root architecture under acid stress, revealing that these microorganisms can modulate root architecture through hormone production, volatile organic compound release, and mineral nutrient regulation (Shen et al., 2025). This underscores the multifaceted role of rhizosphere microorganisms in shaping the rhizosphere environment beyond mere organic matter decomposition. Investigations into rhizosphere microecology under heavy metal and drought stress have also yielded significant insights. Li Na et al. discovered that cadmium stress alters the composition of plant rhizosphere microbial communities (Li et al., 2025). Meanwhile, Gao Yanting et al. explored the microbial carbon source metabolism response to varying drip irrigation levels in arid and semi-arid regions, revealing that moderate and mild water stress treatments can enhance soil microbial carbon source utilization diversity and metabolic capacity (Gao et al., 2022). These studies collectively highlight the intricate interplay between rhizosphere microorganisms, environmental stressors, and plant health.

Light and water constitute indispensable external stimuli for plant growth and development. Recent groundbreaking findings by Professor Xu Jianming have illuminated, for the first time, the phototropic modulation of the circadian rhythm within rice rhizosphere microbial communities (Zhao et al., 2021), demonstrating a pronounced decrease in the *α*-diversity of these microbial assemblages under light exposure. Furthermore, Xie et al. (2023) have observed a marked enhancement in the abundance of rhizosphere microorganisms, particularly those beneficial to plant growth (e.g., lysobacteria and Ramlibacter), along with heightened soil enzyme activity under LED illumination, underscoring the profound influence of light on soil biochemical processes. Concurrently, soil moisture status emerges as a pivotal environmental factor governing soil microbial dynamics and enzymatic activities, as evidenced by numerous studies (Ghezzehei et al., 2019; Siebielec et al., 2020). Notably, Cao Yajing has revealed that while chronic water scarcity prompts microbial adaptation strategies, acute drought stress can lead to microbial cell demise due to loss of resilience (Cao et al., 2023). Under drought conditions, the rhizosphere microbial landscape undergoes profound shifts in diversity, composition, and metabolic activity. Despite these insights, a notable gap persists in rhizosphere microecology research that specifically examines the combined effects of water and light as external factors. The relative significance of these two variables in modulating soil enzyme activities remains elusive. Given the paramount importance of fine-tuning soil enzyme activity and rhizosphere microbial proliferation during plant ontogeny for optimal seedling establishment, this study endeavors to dissect the differential responses of rhizosphere microecosystems in two *Quercus* species seedlings to light and water manipulations. The intricate interplay between these environmental cues, soil enzymes, and rhizosphere microbial communities remains an enigma, necessitating further exploration to unravel their complex relationships.

*Q. dentata* and *Q. variabilis Blume*, two prominent deciduous members of the Fagaceae family, exhibit widespread distribution and multifaceted applications (Liu, 2020; Sun et al., 2023). Their seeds are replete with starch, while their wood and bark are rich sources of tannin, amenable to extraction. In China, *Quercus* species form the backbone of numerous forest communities and occupy a preeminent position among dominant tree species, embodying the virtues of “native adaptability, longevity, stress resilience, food value, and aesthetic appeal.” Their unparalleled contribution to ecological, economic, cultural, and tourism sectors underscores their significance. Within the urban greenscape of Beijing, *Q. variabilis*, as a native species and a recommended tree for urban forestry, holds a pivotal role in the city’s “Underforest Supplementation Oak” initiative, exemplifying exceptional ecological potential (Huang et al., 2022; Zhang et al., 2024). While extensive research has delved into the functional traits, physiological and biochemical characteristics, and photosynthesis of *Q. dentata* and *Q. variabilis* (Qi et al., 2024; Sun et al., 2021; Hao et al., 2020), as well as their seedling cultivation, growth, and development (Cao et al., 2018; Wu et al., 2022; Wang et al., 2022), the intricate interplay between their habitat conditions, soil enzymes, and rhizosphere microbial communities remains an enigma. To unravel this complexity, our study devised a series of 12 experiments, each incorporating distinct water-light gradients, aiming to decipher the dual influence of light and water on the rhizosphere microecology of these two oak species. Leveraging bioinformatics and high-throughput sequencing technologies, we analyzed the dynamic fluctuations in rhizosphere soil enzyme activities and microbial community structures under varying light and water regimes. Our objectives are twofold: (Berendsen et al., 2012) to determine the extent and mechanisms by which light and water conditions impact rhizosphere soil enzyme activities in both oak species; (Birt et al., 2022) to elucidate how light and water conditions reshape the rhizosphere microbial community composition of the seedlings of these two oak species.

## Materials and methods

### Study site and materials

The research was conducted within the confines of the Nanda Wilderness Nursery, situated at 116.19°E, 39.89°N in the Shijingshan District of Beijing. To mimic the diverse understory light regimes in natural settings (Quero et al., 2006), a custom-built shade/canopy structure, spanning 36 square meters, was erected. The primary framework was fabricated from galvanized steel tubes, with transparent rigid plastic sheets serving as the covering for all four light intensity levels. For the three shading treatments, 25, 50, and 75% black shading screen fabrics were incorporated, respectively (Ncise et al., 2020).

This design allowed for flexibility in managing light exposure, with the plastic sheet being readily rolled up during sunny days to optimize light penetration and swiftly secured during rainy periods to shield against precipitation interference, ensuring a controlled environment conducive to experimentation. To maintain optimal ventilation and ensure the integrity of the experimental setup, special attention was paid to the canopy’s ventilation capabilities.

Furthermore, the study employed 1.5-year-old container seedlings of *Q. dentata* and *Q. variabilis*, both of which were bred under uniform provenance conditions and subjected to identical management and protection protocols.

In the experimental setup, a mixed substrate composed of vermiculite and peat in a 1:1 ratio was employed to establish an appropriate growth milieu. The potted seedlings were transferred to the nursery and subjected to a 45-day acclimatization period. During this stage, routine water and fertilizer management protocols were implemented. Watering was conducted at intervals of 3 to 5 days, contingent upon the prevailing weather conditions. Additionally, Osmocote 315 s (at a dosage of 3–5 grams per pot) was applied once to supply the essential nutrient elements, including nitrogen, phosphorus, potassium, etc., requisite for seedling growth. Simultaneously, the fungicide chlorothalonil was sprayed to safeguard against diseases, pests, and fungal and bacterial infections, with applications occurring once every 5 days for a total of 3 repetitions.

Samples seedlings were, respectively, drawn from a total of 120 seedlings under 12 treatment conditions. From this cohort, 36 pots of each species, exhibiting robust growth and free from pests and diseases, were selected to serve as the sample plants for this investigation.

The mean plant height of the potted *Q. dentata* seedlings was quantified at 31.03 cm, concomitant with a basal diameter of 4.31 mm; whereas the mean plant height of the potted *Q. variabilis* seedlings was ascertained to be 42.10 cm, along with a basal diameter of 6.37 mm. The initial growth status of the sample seedlings are detailed in Table 1.

**Table 1:** The initial growth status of the sample seedlings.

Seedling samples	Initial growth status	Mean ± standard deviation
*Q. dentata*	Plant height (cm)	30 ± 1.20
	Basal diameter (mm)	4.2 ± 0.30
	SLA (cm^2^/g)	78 ± 2.20
*Q. variabilis*	Plant height (cm)	41.5 ± 1.50
	Basal diameter (mm)	6.1 ± 0.50
	SLA (cm^2^/g)	80 ± 2.30

### Light and water stress treatment

Four distinct light intensity levels were established: natural light (L0, corresponding to 100% of natural illumination), light shading (L1, 75% of natural light), medium shading (L2, 50% of natural light), and deep shading (L3, 25% of natural light). During seedling growth, the light intensity under varying shade conditions was gauged via the “five-point method.” The sunshade’s configuration was promptly adjusted in accordance with weather changes, guaranteeing that all weather factors, except light intensity, remained relatively uniform across different light treatment zones.

**Figure 1:**
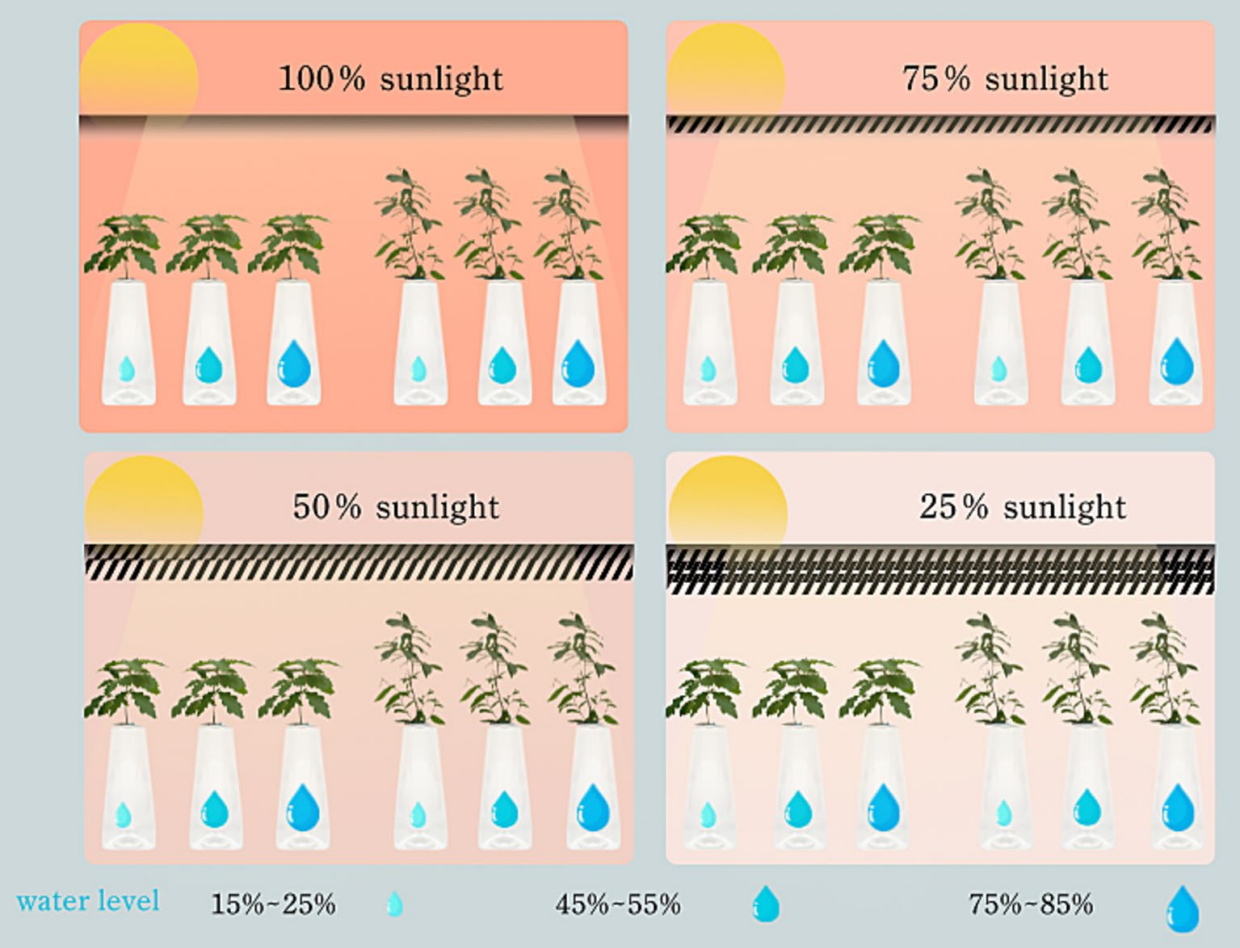
Schematic diagram of experimental design.

To accurately assess and maintain soil water content across these gradients, a combined approach utilizing weighing methods alongside a soil temperature and humidity meter (YM-19-2) was employed. This methodology facilitated the calculation of soil water content and subsequent water supplementation, targeting specific treatment gradients: 75–85% of field water capacity (W0), 45–55% (W1), and 15–25% (W2). Based on prevailing weather conditions, soil water content measurements were conducted every 1–2 days in the evening, with three pots randomly selected from each treatment group. Irrigation was then administered as necessary to adhere to the prescribed water content ranges (Figure 1).

The experimental design adopted a completely randomized block design, ensuring robust statistical analysis and minimizing potential biases. The specific settings and configurations of this experimental setup are detailed in Tables 2, 3.

**Table 2:** Design of the experimental group.

Water control	Light control			
	100%(L0)	75%(L1)	50%(L2)	25%(L3)
75–85% (W0)	L0W0	L1W0	L2W0	L3W0
45–55% (W1)	L0W1	L1W1	L2W1	L3W1
15–25% (W2)	L0W2	L1W2	L2W2	L3W2

**Table 3:** The light intensity under different shading conditions.

Experimental light intensity design	Average midday light intensity (μmol m^−2^ s^−1^)
100%(L0)	1,489
75%(L1)	1,120
50%(L2)	746
25%(L3)	372

### Rhizosphere microbial sample collection and sequencing

In August 2023, we conducted a meticulous sampling of rhizosphere soil from a total of 72 seedlings belonging to two distinct species of *Quercus* plants. Upon reaching the sampling stage, the soil-root matrix within each container was transferred into sterile sampling bags to maintain the integrity and purity of the samples. Upon arrival at the laboratory, a delicate procedure was followed to separate the roots from the soil, which involved gently shaking the roots to dislodge loose soil particles. Subsequently, a sterile brush was employed to collect the residual soil adhering to the roots. The collected rhizosphere soil samples were then passed through a 20-mesh sterile sieve to eliminate larger particulate impurities, ensuring the purity and homogeneity of the samples. The resulting soil samples were promptly subjected to liquid nitrogen quick-freezing for preservation and stored at −80°C in a refrigerator to maintain their integrity until further analysis. From each soil lot, we prepared three replicate samples (yielding a total of 216 small samples), with each individual sample consisting of precisely 0.3 grams of rhizosphere soil. Once the sampling process was completed, all samples were stored in low-temperature conditions to preserve their quality and subsequently dispatched to Shanghai Personalbio Gene Technology Co., Ltd. for comprehensive sequencing analysis.

During the amplicon sequencing analysis, 0.3 g of soil was weighed for each sample. The soil was homogenized, the microorganisms were lysed, and the DNA was isolated and extracted according to the operating instructions in the DNA extraction kit. The purity of the extracted nucleic acid was detected using a NanoDrop micro-spectrophotometer. In addition, 2% agarose gel electrophoresis was employed to examine the integrity of the genomic DNA, to check for degradation and contamination such as proteins. The target fragments were excised and then recovered using the Axygen gel recovery kit. For PCR, the NEB Q5 DNA high-fidelity polymerase was used, and the hypervariable region V3-V4 of the bacterial ribosomal 16S rRNA was selected as the target region for polymerase chain reaction (PCR) amplification. The specific primers for the bacterial 16S rRNA V3-V4 region were 338F (5′-barcode+ACTCCTACGGGAGGCAGCA-3′) and 806R (5’-GGACTACHVGGGTWTCTAAT-3′). The barcode in the forward primer is an oligonucleotide sequence of 7–10 bases, which is used to distinguish different samples in the same library. Similarly, for fungal sequencing, the rDNA ITS sequence analysis technique ITS was utilized for PCR amplification. The ITS1 region primers ITS5 (GGAAGTAAAAGTCGTAACAAGG) and ITS2 (GCTGCGTTCTTCATCGATGC) were selected for amplification and sequencing. The barcode in the forward primer is an oligonucleotide sequence of 7–10 bases, which is used to distinguish different samples in the same library, and the amplification system was the same as that for bacteria.

The PCR products were quantified on a Microplate reader (BioTek, FLx800) using the Quant-iT PicoGreen dsDNA Assay Kit, and then the samples were pooled according to the required data amount for each sample. During the library construction process, the TruSeq Nano DNA LT Library Prep Kit of Ilumina was used for library construction. Firstly, in the end repair process, the End Repair Mix2 in the kit was used to remove the protruding bases at the 5′ end of the DNA, fill in the missing bases at the 3′ end, and add a phosphate group at the 5′ end. Finally, the library was subjected to quality inspection, data quality control, and analysis.

### Soil enzyme activity was determined

To assess the enzyme activity within the rhizosphere soil of two *Quercus* seedlings, we first weighed 0.1–0.5 grams of rhizosphere soil samples, passing them through a 60-mesh sieve to ensure homogeneity. These samples were then transferred into 2-mL centrifuge tubes for subsequent analysis. For the determination of urease activity, the samples were incubated at 37°C for 20 min using the sodium phenol-sodium hypochlorite colorimetric method, following which colorimetric analysis was performed at 578 nm using ultraviolet spectrophotometry, as described by Guan (1986). Similarly, soil catalase activity was determined using a standardized method, with colorimetric readings taken at 240 nm by ultraviolet spectrophotometry, adhering to the protocol outlined by Yang L. F. et al. (2011). For the quantification of soil sucrase and cellulase activities, we employed the 3,5-dinitrosalicylic acid colorimetric method, with colorimetric measurements conducted at 540 nm using ultraviolet spectrophotometry. Meanwhile, soil acid phosphatase activity was determined using the phenylene disodium phosphate colorimetric method, with colorimetric readings taken at 400 nm, as detailed by Li et al. (2008). Lastly, soil chitinase activity was measured by a specific method, with colorimetric analysis performed at 544 nm using ultraviolet spectrophotometry, according to the guidelines provided by Gu and Hu (1994). These rigorous methodologies ensured accurate and reliable assessments of the various enzyme activities present in the rhizosphere soil of the *Quercus* seedlings.

### Data analysis

Experimental data integration and processing were facilitated using Microsoft Excel 2020 software, while statistical analyses were conducted with SPSS 27.0 software (SPSS, Chicago, IL). To investigate the responses of six soil enzymes in the rhizosphere of *Q. dentata* and *Q. variabilis* seedlings to varying water and light conditions, a two-way ANOVA was employed. For data visualization, Origin 2017, R version 4.1.2, and Python programming languages were utilized. Regarding microbiome bioinformatics, QIIME2 (version 2019.4) served as the primary tool for analysis. Primer fragments were excised using cutadapt, and subsequent steps including quality control, noise reduction, sequence assembly, and chimera removal were executed with DADA2. ASV (Amplicon Sequence Variants) feature sequences and tables were merged, and each ASV’s feature sequence was classified using QIIME2’s classify-sklearn algorithm, leveraging a Naive Bayes classifier against the Greengenes database for species annotation. To ensure consistent sample depth, the ASV table was rarefied to 95% of the minimum sample sequence size using QIIME2’s qiime feature-table rarefy function. All subsequent analyses were conducted on this rarefied ASV table, providing a robust foundation for our ecological insights.

## Results

### Effects of light and water manipulation on rhizosphere soil enzyme activities in seedling growth environments

**Figure 2:**
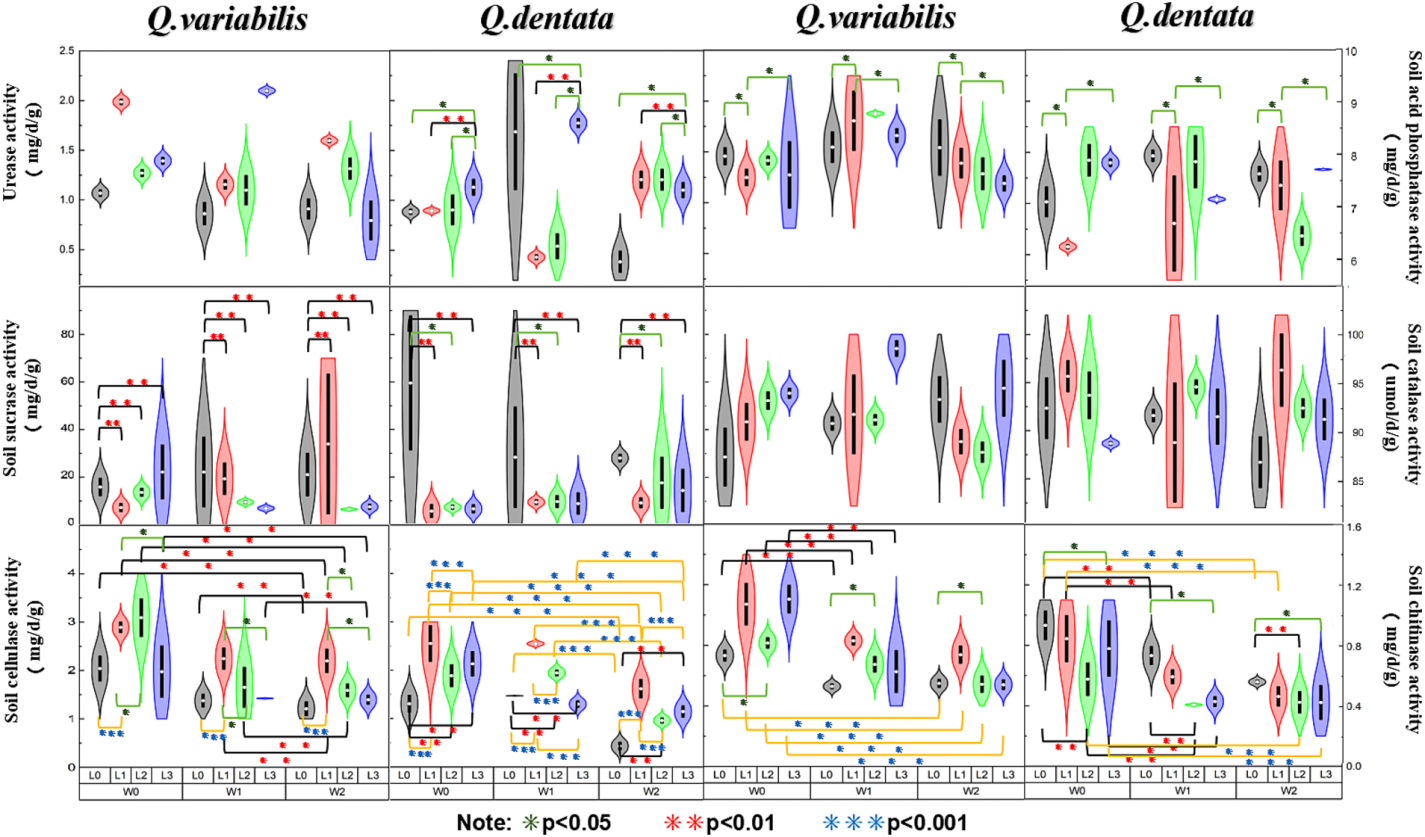
Soil enzyme activities among different seedling growth environments (*p < 0.05, **p < 0.01, ***p < 0.001).

The results presented in Figure 2 reveal several key findings regarding the enzymatic activities in the rhizosphere soil of *Q. dentata* and *Q. variabilis*. Specifically, the urease activity in the rhizosphere soil of *Q. dentata* was significantly influenced by light (*p <* 0.05) and the interaction between water and light (*p <* 0.01). In contrast, the urease activity in the rhizosphere soil of *Q. variabilis* exhibited significant differences under varying light (*p <* 0.001) and water-light interaction (*p <* 0.001) conditions, as well as under different water conditions *(p <* 0.01). The activity of acid phosphatase in the rhizosphere soil of *Q. dentata* was notably affected by the interaction between water and light (*p <* 0.05), whereas in *Q. variabilis*, this activity showed significant differences under different water conditions (*p <* 0.05). The activities of sucrase in the rhizosphere soil of both Quercus seedlings were not significantly impacted by water and light (*p* > 0.05). Within the L2 group, the sucrase activities in the rhizosphere soil of *Q. variabilis* seedlings ranged from 4.56 mg/d/g to 63.36 mg/d/g, while those in *Q. dentata* ranged from 2.94 mg/d/g to 87.80 mg/d/g, the range of sucrase activity in the rhizosphere of the same type of seedlings was relatively large.

The catalase activity in the rhizosphere soil of *Q. variabilis* showed significant differences under different light conditions (*p <* 0.05), whereas the influence of water on catalase activity in both *Quercus* species was not significant. Both water and light conditions had significant effects on the cellulase activity in the rhizosphere soil of *Q. dentata* and *Q. variabilis* seedlings (*p <* 0.01), with an extremely significant effect observed in *Q. dentata* seedlings (*p <* 0.001). Lastly, the chitinase activity in the rhizosphere soil of both *Quercus* plants was significantly influenced by light (*p <* 0.05) and water (*p <* 0.001).

### Structure and composition of rhizosphere microbial community

The microbial diversity associated with *Q. dentata* exhibited the lowest levels under the L1W0 conditions (75–85% water, 75% light), encompassing 5 phyla, 11 classes, 21 orders, 27 families, 33 genera, and 37 species of fungi, as well as 21 phyla, 40 classes, 87 orders, 119 families, and 145 genera of bacteria. Notably, an identical claim of minimal diversity was inadvertently repeated for L2W0 conditions, which should be corrected to reflect unique findings if applicable; however, for the sake of this revision, we will focus on the intended comparison. Under the 50% light condition, the microbial diversity peaked, with a comprehensive tally of 6 phyla, 11 classes, 24 orders, 29 families, 48 genera, and 56 species of fungi, alongside 23 phyla, 48 classes, 97 orders, 136 families, and 159 genera of bacteria.

**Figure 3:**
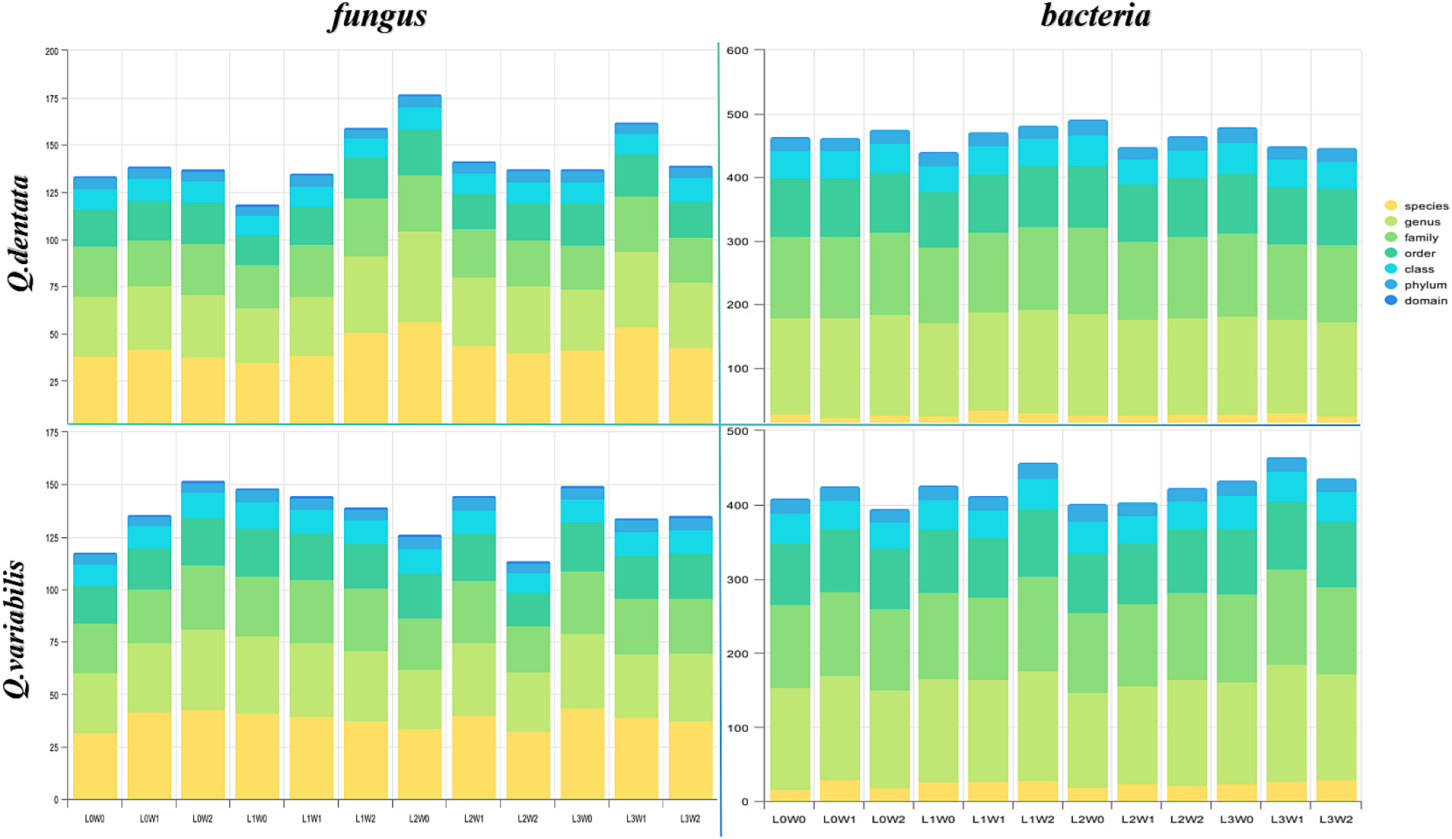
The number of taxa of rhizosphere microorganisms.

Among the 12 treatments examined, the highest fungal diversity was observed under L0W2 conditions (15–25% water, 100% light), featuring 4 phyla, 12 classes, 22 orders, 30 families, 38 genera, and 43 species of fungi. Conversely, bacterial diversity reached its zenith under L3W1 conditions (45–55% water, 25% light). Under L2W2 conditions (15–25% water, 50% light), a rich bacterial assemblage of 19 phyla, 40 classes, 91 orders, 128 families, 158 genera, and 27 species coexisted with a fungal community comprising 4 phyla, 9 classes, 15 orders, 22 families, 28 genera, and 32 species. Under full light exposure (100% light), bacterial diversity was notably depleted, consisting of 17 phyla, 35 classes, 81 orders, 109 families, 132 genera, and only 18 species. When comparing the rhizosphere bacteria of *Q. variabilis* and *Q. dentata*, the former demonstrated greater adaptability to drought conditions, whereas the latter exhibited a higher overall bacterial diversity (Figure 3).

### Rhizosphere microorganisms diversity

**Figure 4:**
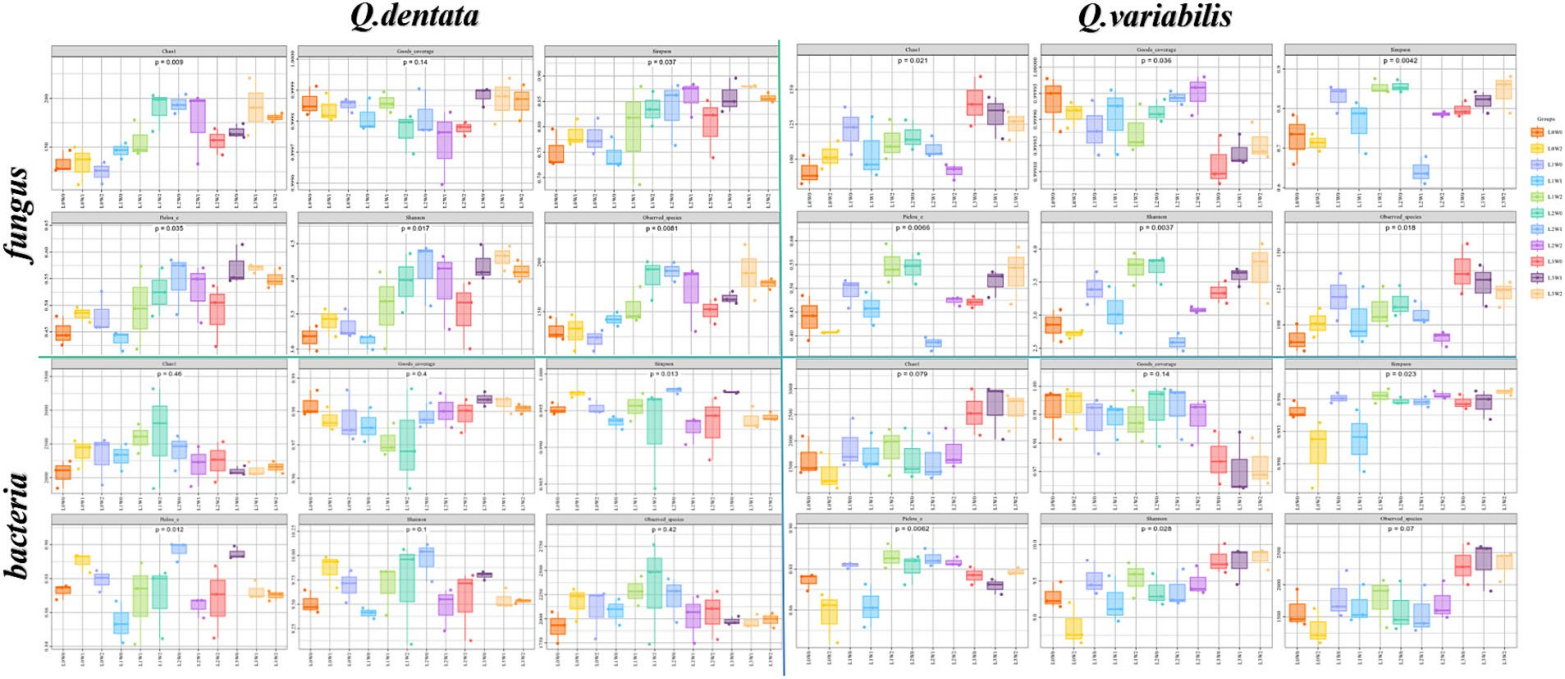
Rhizosphere microbial Alpha diversity index.

The results indicate significant variations in fungal and bacterial communities within the rhizosphere soil of both *Q. dentata* and *Q. variabilis* in response to different light and water treatments. Specifically, for *Q. dentata*, the Chao 1 (*p <* 0.01) and Simpson (*p <* 0.05) indices revealed that fungi exposed to the L1 light treatment exhibited significantly higher abundance compared to those under the L0 treatment, with the L1W2 treatment yielding the highest fungal richness. This suggests that fungi in the rhizosphere soil of *Q. dentata* thrive better under L1 light conditions. Furthermore, in the Pielou’s evenness index (*p <* 0.05), the highest distribution uniformity was observed under L2W0 conditions (75–85% water, 50% light), which distinctly differed from other treatments. Notably, for bacteria, the L2W0 conditions also demonstrated the highest diversity and most uniform community distribution in both the Simpson (*p <* 0.05) and Pielou (*p <* 0.05) indices, with these differences being more pronounced compared to other treatments. However, in the Chao 1 index (*p* > 0.05), no significant difference was observed in rhizosphere soil bacterial community richness (Figure 4).

In the case of *Q. variabilis*, the Chao 1 index (*p <* 0.05) showed that fungal richness peaked under the L3 light condition, with the L3W0 treatment exhibiting the most significant difference compared to others. The Simpson index (*p <* 0.01) further confirmed that the L1 light treatment significantly outperformed the L0 treatment, while other treatments also showed significant differences. Additionally, the Pielou’s evenness index (*p <* 0.01) indicated that the distribution uniformity of fungi in rhizosphere soil was significantly influenced by both light and water, with notable differences observed between L2W0 (75–85% water, 50% light), L2W1 (45–55% water, 50% light), L3W2 (15–25% water, 25% light), L3W0, and other treatments. Analysis of bacterial groups revealed substantial bacterial diversity in the rhizosphere under L2 and L3 light conditions in the Simpson index (*p <* 0.05), with significant differences compared to L0W2 (15–25% water, 100% light) and L1W1 (45–55% water, 75% light). However, in the Shannon index (*p <* 0.05), L3 exhibited the greatest bacterial diversity under light conditions, with significant differences from other treatments (Figure 4).

**Figure 5:**
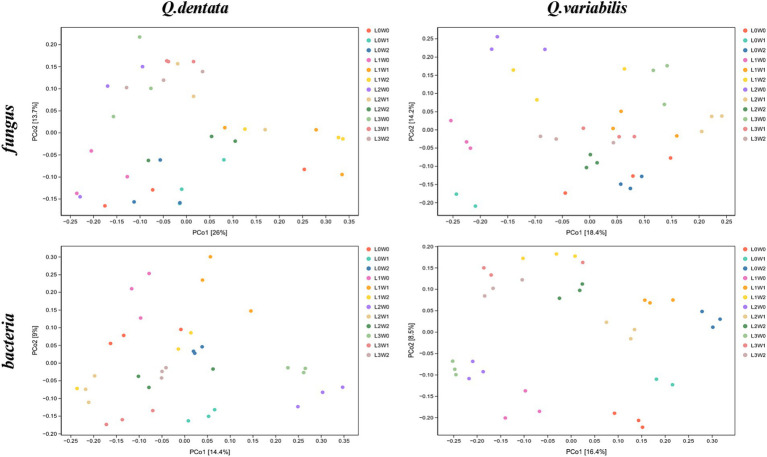
PCoA analysis of rhizosphere microorganisms.

The Principal Coordinates Analysis (PCoA) diagrams provide further insights into the bacterial community compositions. The first axis of the PCoA diagram for *Q. dentata* explains 14.4% of the inter-sample differences, while the second axis accounts for 9%. Visual inspection of the diagram reveals substantial differences among bacterial communities from treatments such as L1W1, L0W1, L2W1, L3W1, L0W0, L1W0, L2W0, and L3W0. Similarly, for *Q. variabilis*, the first axis of the PCoA diagram explains 16.4% of the inter-sample differences, and the second axis explains 8.5% (Figure 5).

### Comparative analysis of rhizosphere fungal species differences

**Figure 6:**
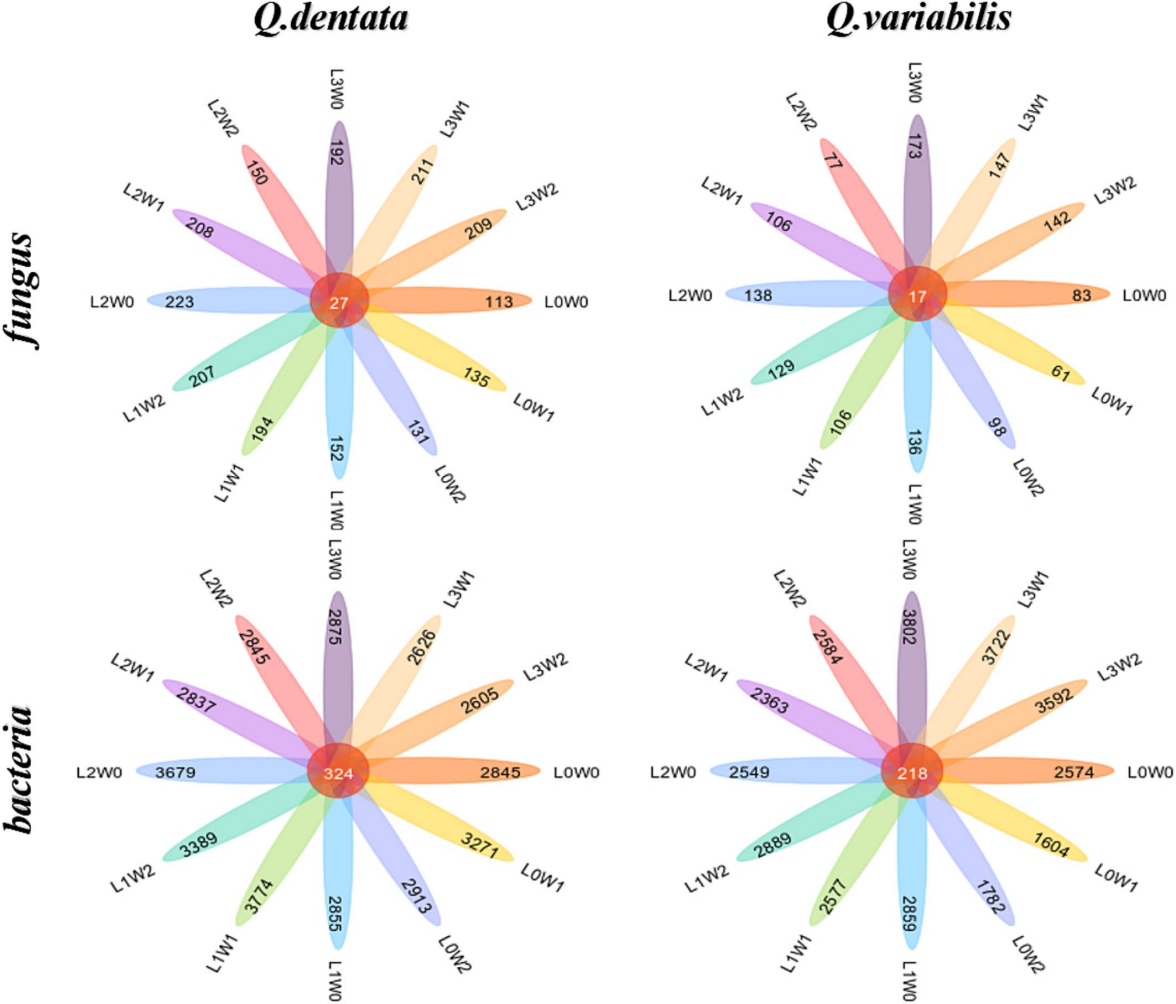
Wayne diagram of rhizosphere soil microbial ASV under different treatments.

The species difference analysis of rhizosphere fungal communities was conducted for two *Quercus* seedlings, with the Wayne diagram analysis revealing that 27 ASVs in the rhizosphere soil of *Q. dentata* were common across all 12 treatment groups, whereas 17 ASV species in the rhizosphere soil of *Q. variabilis* were similarly shared among these groups (Figure 6). Notably, the number of bacterial species in the rhizosphere significantly outnumbered the fungal species. Analysis of rhizosphere bacterial communities indicated that 324 ASV species were shared among the 12 treatment groups in the rhizosphere soil of both seedlings, with 218 ASVs commonly found in the rhizosphere soil of *Q. variabilis* (Figure 6).

**Figure 7:**
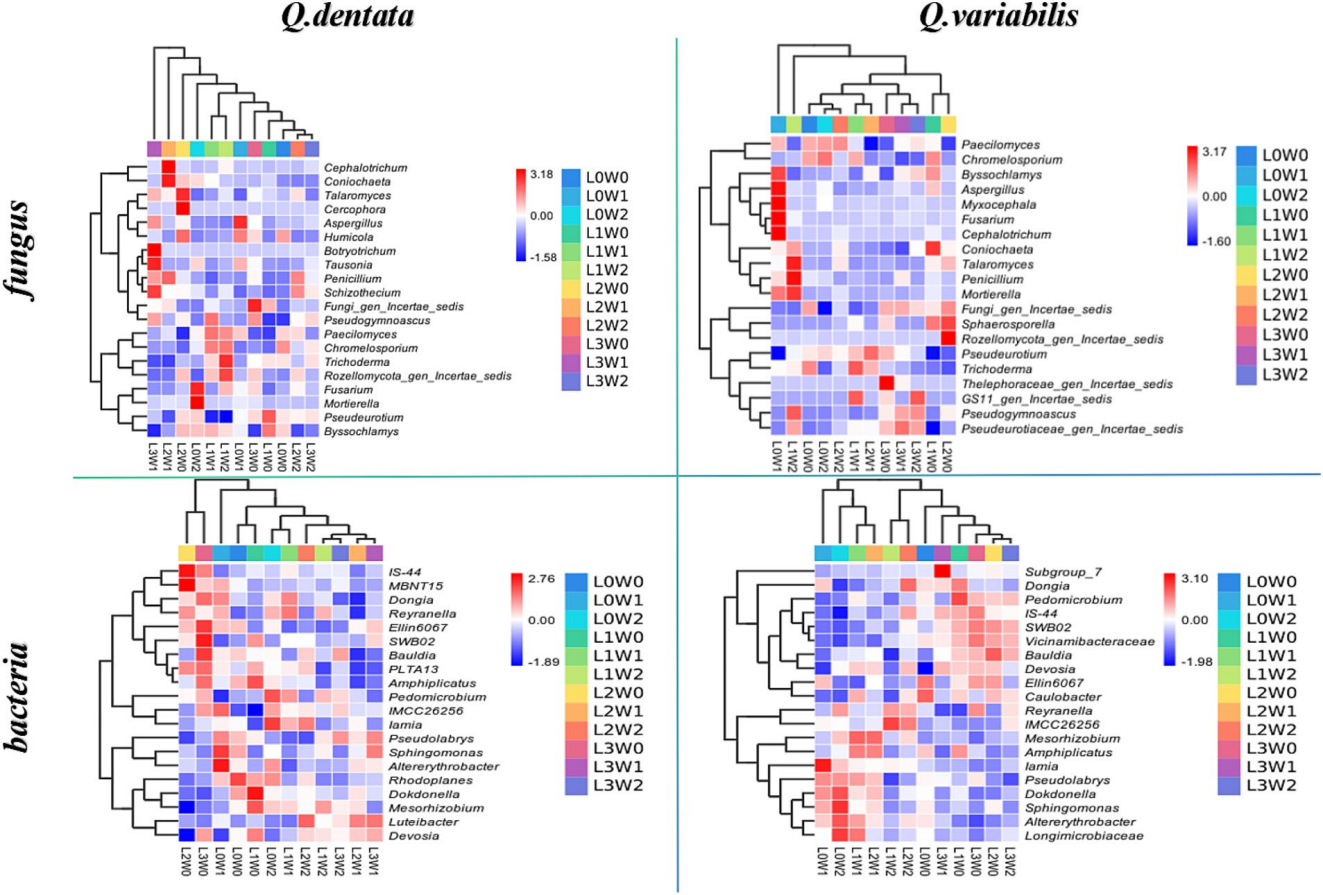
Heat map of rhizosphere soil microbial species composition under different treatments.

To further compare species composition among the samples and visualize the trend of species abundance distribution, heat maps were generated using abundance data for the top 20 genera with the highest average abundance. Additionally, UPGMA clustering of the samples was performed based on the Euclidean distance of species composition data. The results demonstrated that species such as *Cercospora*, *Botryotrichum*, *Frankia*, and *Sphingobium* exhibited minimal variation under different treatments. Conversely, significant differences were observed among the microbiomes of Rozellomycota, *Penicillium*, *Trichoderma*, *Streptomyces*, *Mesorhizobium*, and Gemmatimonas. Specifically, the rhizospheres of *Q. variabilis* (with respect to Gemmatimonas), Fusarium (with respect to Fusarium), and *Cephalotrichum* (with respect to *Cephalotrichum*) showed limited differences under various treatments. However, significant variations were noted for *Penicillium*, *Streptomyces*, *Mesorhizobium*, *Methylobacterium*, and other microbiomes (Figure 7).

**Figure 8:**
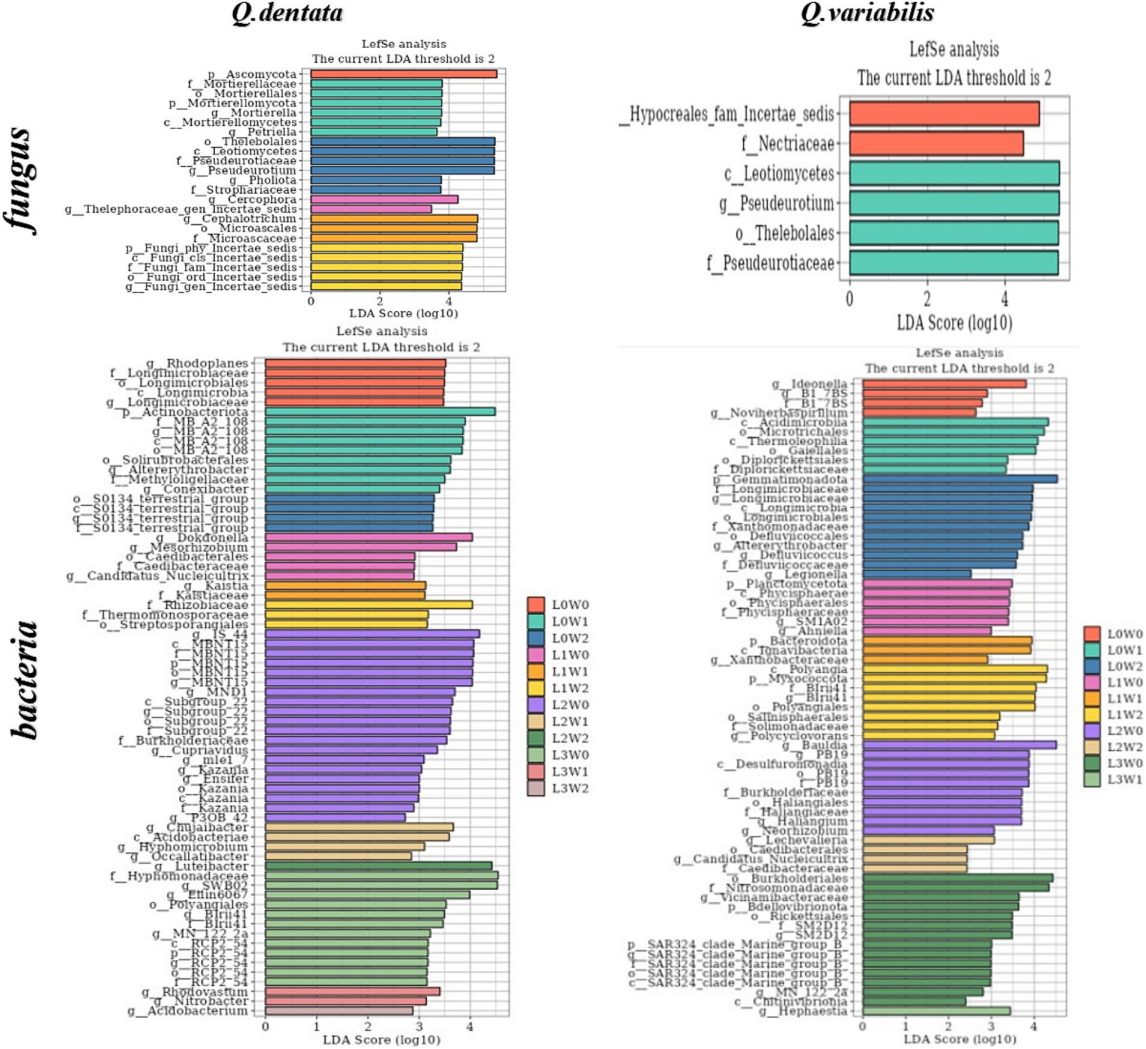
Histogram of rhizosphere microbial LDA.

An LDA threshold of 2 was established, such that only species surpassing this threshold were deemed biomarker species with significantly higher abundance compared to other groups. The LDA histogram depicted the distinct rhizosphere microbial species in seedlings of *Q. dentata* and *Q. variabilis* under 12 water and light condition groups. The results highlighted significant differences in rhizosphere soil microbial composition between the two *Quercus* seedlings under different treatments. Notably, Ascomycota was found in the rhizosphere of *Q. dentata* under L0W0 (100% light, 75–85% water) treatment, while Nectriaceae fungi, Acidimicrobiia bacteria, and actinomycetes were detected in the rhizosphere of *Q. variabilis* under L0W1 (100% light, 45–55% water) treatment. Under L1W0 (75% light, 75–85% water) treatment, Thelebolales fungi and Leotiomycetes fungi were identified in the rhizosphere of *Q. variabilis*. Additionally, *Cephalotrichum* and Microascales were observed in the rhizosphere of *Q. dentata* under L2W1 (50% light, 45–55% water) treatment, while Gemmatimonadetes was found in the rhizosphere of *Q. variabilis* under L0W2 (100% light, 15–25% water) treatment. Furthermore, Bauldia was detected in the rhizosphere of *Q. variabilis* under L2W0 (50% light, 75–85% water) treatment, and Luteibacter was found in the rhizosphere of *Q. dentata* under L2W2 (50% light, 15–25% water) treatment. Under L3W0 (25% light, 75–85% moisture) treatment, Hyphomonadaceae and SWB02 were identified in the rhizosphere of *Q. dentata*, while the effect value of microorganisms such as Burkholderiales and Nitrosomonadaceae in the rhizosphere of *Q. variabilis* exceeded 4, indicating these microorganisms as significantly different species among the samples (Figure 8).

## Discussion and conclusion

### Influence of light and water conditions on enzyme activities in oak seedling rhizosphere soil

Previous research on soil enzyme activities has predominantly emphasized the input of nutrients, particularly nitrogen and phosphorus. However, our focus shifts to the rhizosphere microenvironment, which serves as a dynamic and highly active zone for soil microorganisms. This microenvironment exhibits a marked responsiveness to alterations in the soil milieu, and this sensitivity is manifested through alterations in enzyme activities (Zhang et al., 2014). Notably, soil enzymes not only exert a direct influence on plant growth but can also indirectly impact plant health by disrupting the plant’s antioxidant system (Lian et al., 2024). Our findings align with these observations, indicating that the rhizosphere’s response to environmental cues is intricately linked to soil enzyme activities, which in turn regulate plant growth and resilience. However, it is important to note that not all microorganisms are susceptible to drought conditions. In fact, some microorganisms demonstrate remarkable drought resistance, which can alter the overall activity of extracellular enzymes in soil (McDaniel et al., 2013; Zuccarini et al., 2023). In specific instances, a compensatory mechanism arises between plants and microorganisms in response to drought stress. This mechanism helps to mitigate the detrimental effects of drought on extracellular enzymes, thereby bolstering the resilience of the entire ecosystem (Preece and Penuelas, 2016).

Several studies have highlighted that soil urease is not only a pivotal enzyme for urea hydrolysis but also possesses heat stability (Wang and Huang, 2003). Our study revealed that the activity of this enzyme is significantly influenced by light and water conditions. Specifically, excessive light may expedite the decomposition of urease, disrupting its structure, reducing its concentration, and consequently weakening its activity. These findings align with the research conducted by Cai et al. (2016), which observed significant differences in urease activity in the rhizosphere soil of *Q. variabilis* under varying light and water-light interaction conditions. Notably, compared to *Q. dentata*, the urease activity in the rhizosphere soil of *Q. variabilis* sinensis seedlings exhibited greater sensitivity to changes in water and light. Given the strong correlation between urease activity and soil nitrogen content, which facilitates the conversion of urea into ammonia (Craine et al., 2009), variations in urease activity serve as a reliable indicator of the intensity of soil nitrogen transformation processes (Tan, 2024).

Acid phosphatase (ACP) is a ubiquitous hydrolase in plants that plays a crucial role in phosphorus absorption and utilization (Zhao X. W. et al., 2020; Hu et al., 2024). Prior studies have shown that ACP activity is influenced by the plant’s phosphorus supply status (Song et al., 1999; Dracup et al., 1984). Our study revealed that the response of ACP in the rhizosphere soil of two *Quercus* species to changes in external environmental factors varied. Specifically, ACP activity in the rhizosphere soil of *Q. dentata* was affected by both light and water, whereas ACP activity in the rhizosphere soil of *Q. variabilis* was more significantly influenced by water alone. This difference may be attributed to the rhizosphere effect of *Q. dentata* seedlings, which is produced under the dual influence of light and water (Figure 2). The rhizosphere effect results in higher enzyme activity in rhizosphere soil compared to non-rhizosphere soil, and the presence of numerous microorganisms around plant roots facilitates the enhancement of phosphatase activity in rhizosphere soil (Spohn et al., 2015).

Soil sucrase is directly involved in the soil carbon cycle and is a key enzyme that catalyzes sucrose hydrolysis. It is commonly used as an indicator of soil carbon nutrition status (Chioti and Zervoudakis, 2017), and changes in its activity can reflect the activity of the soil carbon cycle (Li et al., 2005). Studies have shown that sucrase activity is significantly or extremely significantly correlated with most soil factors and the contents of nitrogen, phosphorus, and potassium in plants. In this study, we explored changes in sucrase activity in the rhizosphere soil of plants from the perspective of external environmental factors (light and water). The results indicated that there was no significant difference in sucrase activity in the rhizosphere soil of the two *Quercus* seedlings under different water and light conditions. However, the range of activity within each treatment group was large, which may be related to other external factors. This stability may be due to the regulation of the *Q. dentata* and *Q. variabilis* root systems, which maintain the pH and organic matter content of the rhizosphere soil within a relatively stable range, thereby ensuring the stability of sucrose content (Figure 2). Soil catalase is a key enzyme that can decompose soil hydrogen peroxide and is one of the key enzymes in the biological defense system, and its activity is closely related to soil respiration and microbial activities (Yang et al., 2019). In this study, it was found that light conditions had a significant effect on catalase activity, while water had no significant effect on it. The catalase activity of two kinds of *Quercus* seedlings was the highest under L3 (25% light) and L1 (75% light) treatment, respectively, which may be because light affects soil temperature and root secretion production (Figure 2). The change of temperature and secretion will affect the catalase activity in the soil, and the decrease of catalase activity means the decrease of the REDOX buffer capacity of the soil and the change of the REDOX state of the soil rhizosphere, which is not conducive to the absorption and transformation of plant nutrient elements. Some studies have shown that catalase activity increases under strong light and dark conditions, indicating that the antioxidant system is activated under high light stress (Gan et al., 2012), which is consistent with the results obtained in this study.

Soil cellulase (SCL) is a complex enzyme that acts as a biocatalyst in the decomposition of cellulose-containing litter (Xiao D. et al., 2015; Xiao Y. et al., 2015). Its activity is of significant scientific importance in characterizing the degree of soil maturation (Xu et al., 2022). Previous studies have indicated that a reduction in light intensity can influence soil enzyme activity by affecting soil moisture content (SMC), pH, and nutrient levels (Liu et al., 2022). By manipulating external environmental factors such as water and light, this study found that both factors had notable effects on the activity of cellulase in the rhizosphere soil of *Q. dentata* and *Q. variabilis* seedlings (Figure 2). Specifically, the differences in cellulase activity in the rhizosphere soil of *Q. dentata* seedlings were more pronounced under varying water and light conditions compared to *Q. variabilis* (Figure 2). Irrigation water can stimulate the growth and metabolism of soil microorganisms, thereby enhancing the activity of cellulase.

Chitin, a nitrogen-containing polysaccharide, is widely distributed in nature and is second only to cellulose in abundance (Shen, 2021). Chitinase decomposes chitin to release nutrients such as nitrogen, phosphorus, and potassium, which are beneficial for plant absorption and utilization. This process not only improves soil quality and fertility but also promotes plant growth and development, ultimately enhancing crop yields. Some studies have shown that soil water content is closely linked to enzyme activity, and optimal water content can enhance its activity (Li et al., 2018), which aligns with the findings of this study. Our study revealed that chitinase activity in the rhizosphere soil of both *Quercus* species was significantly influenced by light and water. As soil irrigation water decreased, soil moisture content diminished, inhibiting soil enzyme function and naturally reducing its activity (Zhu L. et al., 2021). This further suggests that chitinase thrives better in moist soil environments.

Our study demonstrated that soil enzymes exhibit optimal activity within a specific temperature range. Beyond this range, their activity may be weakened or even completely lost. Light and water, as crucial environmental factors, indirectly influence soil enzyme activity by modulating soil aeration, temperature, and the activity of soil microorganisms. Notably, rhizosphere soil enzyme activities of different plant species respond uniquely to external environmental factors. These findings not only underscore the sensitivity and adaptability of soil enzymes to environmental changes within the plant rhizosphere microenvironment but also offer valuable insights for further elucidating the role of soil enzymes in facilitating plant growth and development.

### Role of light and water conditions in shaping the microbial community in oak seedling rhizosphere soil

Research has indicated that numerous soil properties, including pH, salinity, temperature, and humidity, significantly influence the composition of microbial communities in intricate soil matrices (Frindte et al., 2019; Zheng et al., 2019). Furthermore, temporal variations such as weather patterns, root exudation, and seasonal inputs of organic matter can also affect the structure and activity of these microbial communities (Kuzyakov and Evgenia, 2015; Chernov and Zhelezova, 2020). Soil microbial communities play a crucial role in decomposing soil organic matter, enhancing nutrient storage conditions, and facilitating nutrient cycling through metabolic processes. These processes serve as direct indicators of soil microecological status and disease progression trends, and they represent sensitive early warning signals of soil ecosystem health (Dong et al., 2016; Zuppinger-Dingley et al., 2014). Notably, the diversity of soil microbial communities responds variably to environmental disturbances (Cai et al., 2020), aligning with the findings of the present study. Regarding the impact of water and light conditions on rhizosphere microbial community dynamics in seedlings, this study revealed that under varying water and light treatments, the microbial community richness, diversity, species abundance, and distribution uniformity of two *Quercus* species underwent significant alterations (Figures 3–5). Notably, there appears to be a substantial correlation between microbial diversity and soil enzyme activity, which is pivotal in maintaining ecosystem multifunctionality (Delgado-Baquerizo et al., 2017). In this study, fungi exhibited greater sensitivity to external light and water treatment conditions compared to bacteria. This may be attributed to the physiological adaptability and community structural differences between fungi and bacteria, resulting in their varied responsiveness to light and water. Fungi generally prefer dark and humid environments, whereas bacteria may exhibit broader adaptability (Figure 6). Consequently, changes in external light and water conditions may more profoundly affect the fungal community. Fungi are typically associated with the decomposition of C- and N-poor substrates, whereas bacteria are more sensitive to unstable substrates (Treseder et al., 2016; Xu et al., 2015). Intermediate decomposition products produced by fungi can serve as unstable materials for bacteria (Romaní et al., 2006). An increased abundance of microorganisms in the rhizosphere soil accelerates the decomposition rate of organic matter, providing more nutrients for seedling growth, thereby promoting their development.

Biodiversity is an intricate concept encompassing various components, such as species richness (the number of taxa) and composition (the identity and relative abundance of the organisms comprising the community) (Diaz and Cabido, 2001), among others. In this study, we observed that the fungal diversity in the rhizosphere soil of the two *Quercus* species was notably lower compared to bacterial diversity. Furthermore, the rhizosphere microbial communities of each sample exhibited high uniqueness and low similarity. At the phylum level, *Ascomycota* dominated the rhizosphere fungal communities of both *Quercus* seedlings, occupying a substantial proportion (Figure 7). Conversely, the rhizosphere bacterial communities were primarily composed of Proteobacteria, *Actinobacteriota*, and *Acidobacteriota*, with *Proteobacteria* being the most abundant. Whole genome sequencing (Trivedi et al., 2013) has provided insights into the distinct roles of Proteobacteria and *Actinobacteriota* in supporting crucial ecosystem processes like decomposition and nutrient cycling. Based on these findings, it can be deduced that Ascomycota and another fungal group play pivotal roles in the growth and development of plant seedlings. Notably, there were significant differences in the abundance of *Paecilomyces*, *Streptomyces*, and *Mesorhizobium* in the rhizosphere soil of *Q. dentata* and *Q. variabilis*, suggesting that these microorganisms are more sensitive to changes in light and water conditions compared to others.

Regarding the microbial community markers and species differences among samples, our findings revealed that the fungal composition in the rhizosphere soil of the two *Quercus* seedlings varied significantly under different treatments, albeit with fewer samples exhibiting differences compared to bacteria. Specifically, *Ascomycetes*, *Trichospora*, *Microcomycetes*, and *Actinomycetes* in the rhizosphere of *Q. dentata*, as well as *Leotiomycetes*, *Acidomycetes*, *Burkella*, *Nitrosomonas*, and other microflora in the rhizosphere of *Q. variabilis*, were notably distinct species. Notably, even seedlings of the same plant species exhibited significant differences in the dominant microbial species within their rhizosphere soil under varying light and water treatments. This could be attributed to microorganisms’ ability to adapt to different environments by modifying their metabolic pathways and growth strategies, thereby establishing dominance in the rhizosphere soil. For instance, as light conditions diminished, the abundance of rhizosphere bacteria and four physiological groups of bacterial grasses decreased, whereas the fungal population gradually increased, leading to a shift in the rhizosphere soil from a “bacterial-dominated” to a “fungal-dominated” type (Forey et al., 2015).

The results indicated that intense light hindered the growth of rhizosphere bacteria, whereas optimal light and humidity conditions fostered more vigorous rhizosphere bacterial community growth, suggesting that these environmental factors significantly influence rhizosphere bacterial flora (Hou et al., 2021; Zhang et al., 2022). Additionally, the rhizosphere bacteria of *Q. variabilis* exhibited greater drought tolerance, providing a survival advantage in arid environments. Although the rhizosphere bacteria of *Q. dentata* showed slightly less drought tolerance, their higher bacterial diversity implied more flexible adaptations to environmental stress. Furthermore, the increased water requirements of *Q. variabilis* under low light conditions likely helped mitigate the slower root growth resulting from insufficient light, emphasizing the crucial role of water in plant growth. On the other hand, fungal communities exhibited distinct responses to environmental conditions compared to bacteria. The fungal community was significantly impacted by light and water, and moderate shade treatment increased fungal species diversity (Yang D. et al., 2011), indicating fungi’s sensitivity to environmental changes. During the seedling stage, the types of bacteria in the rhizosphere microorganisms of *Q. dentata* and *Q. variabilis* showed some similarity under the dual influence of water and light, whereas the fungal types differed significantly (Figure 8). This might be attributed to the specific tendencies of plants at the seedling stage to demand and select microorganisms during their growth process. The variation in the abundance of rhizosphere microorganisms is influenced not only by microbial interactions but also by the external environment and the plant’s growth stage, highlighting the need to consider the role of multiple factors comprehensively when studying rhizosphere microorganisms.

## Data Availability

The raw data supporting the conclusions of this article will be made available by the authors, without undue reservation.
